# Food Insecurity and Mental Health Trajectories During the COVID-19 Pandemic: Longitudinal Evidence

**DOI:** 10.1093/geroni/igab046.1578

**Published:** 2021-12-17

**Authors:** Dexia Kong, Peiyi Lu, Elissa Kozlov, Mack Shelley

**Affiliations:** 1 The Chinese University of Hong Kong, Hong Kong , Hong Kong; 2 Columbia University, Fort Lee, New Jersey, United States; 3 Rutgers University, Piscataway, New Jersey, United States; 4 Iowa State University, Ames, Iowa, United States

## Abstract

The extent to which food insecurity impacts changes in mental health outcomes over time in the context of Covid-19 remains unknown. Using longitudinal data from a nationally representative survey, the objectives of the present study were to: (1) assess the prevalence of food insecurity among U.S. adults amid the Covid-19 pandemic; and (2) investigate the relationships between food insecurity statuses and changes in mental health outcomes over time as the pandemic unfolds. Longitudinal data from the Internet-based Understanding Coronavirus in America survey collected bi-weekly between April and December 2020 were used (n=4,068, 15 repeated measures). Adult respondents (aged ≥18) were asked about their food insecurity experiences and stress/anxiety/depressive symptoms. Linear mixed-effect models examined changes in mental health outcomes over time among groups with various food insecurity statuses. Overall prevalence of food insecurity was 8%. Food insecurity was consistently associated with higher levels of stress/anxiety/depressive symptoms (p<0.001). Stress/anxiety/depressive symptoms declined over time among food-secured U.S adults. However, mental health trajectories of respondents with various food insecurity categories, including food insecurity status, persistent food insecurity, and food insecurity of higher severity and longer duration, remained stable or worsened over time. Moreover, the mental health gap between food-secured and food-unsecured participants widened over time. Food insecurity represents a pressing public health problem during the Covid-19 pandemic with substantial mental health implications. Persistent and severe food insecurity may contribute to mental health disparity in the long term. Food insecurity reduction interventions may alleviate the estimated alarming mental health burden as the pandemic unfolds.

